# Supplementary material for: Gluten Exorphins Promote Cell Proliferation through the Activation of Mitogenic and Pro-Survival Pathways
Source: Int J Mol Sci. 2023 Feb 15;24(4):3912. doi: 10.3390/ijms24043912 (PMC9966116; doi:10.3390/ijms24043912)
Supplement: Supplementary file 1 [file ijms-24-03912-s001.zip › ijms-2173573-supplementary.pdf]

SUPPLEMENTARY MATERIAL

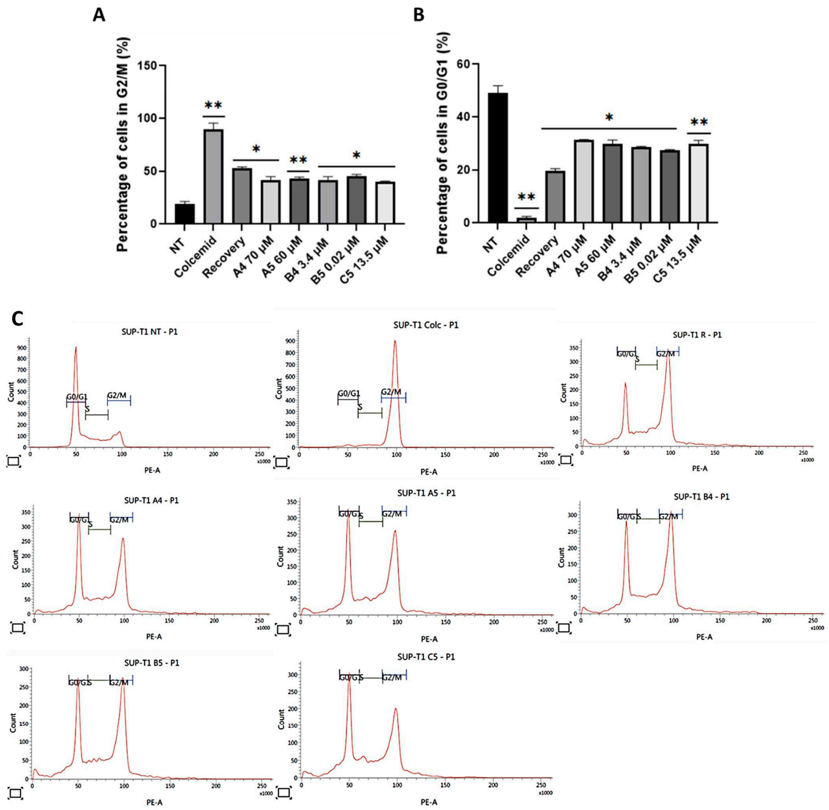

Figure S1. Cytofluorimetric analysis of cell cycle progression in synchronized SUP-T1 cells treated with GEs. GEs were used according to the concentrations reported in the BIOPEP database. Cytofluorimetric analysis was performed at 24 hours p.t. by means of FACS BD Lyrics instrument. Analysis was performed on independent biological replicas (n=3) with 10,000 collected events each. Histograms (A,B) representing the percentage of cells in G2/M (A) and G0/G1 (B) cell cycle phases after GEs treatment are reported as mean ± SEM. Asterisks indicated $p<0.05$ (*) or $p<0.01$ (**), compared with sample R, according to ANOVA One-Way. The relative cytofluorimetric plot are reported below (**C**).

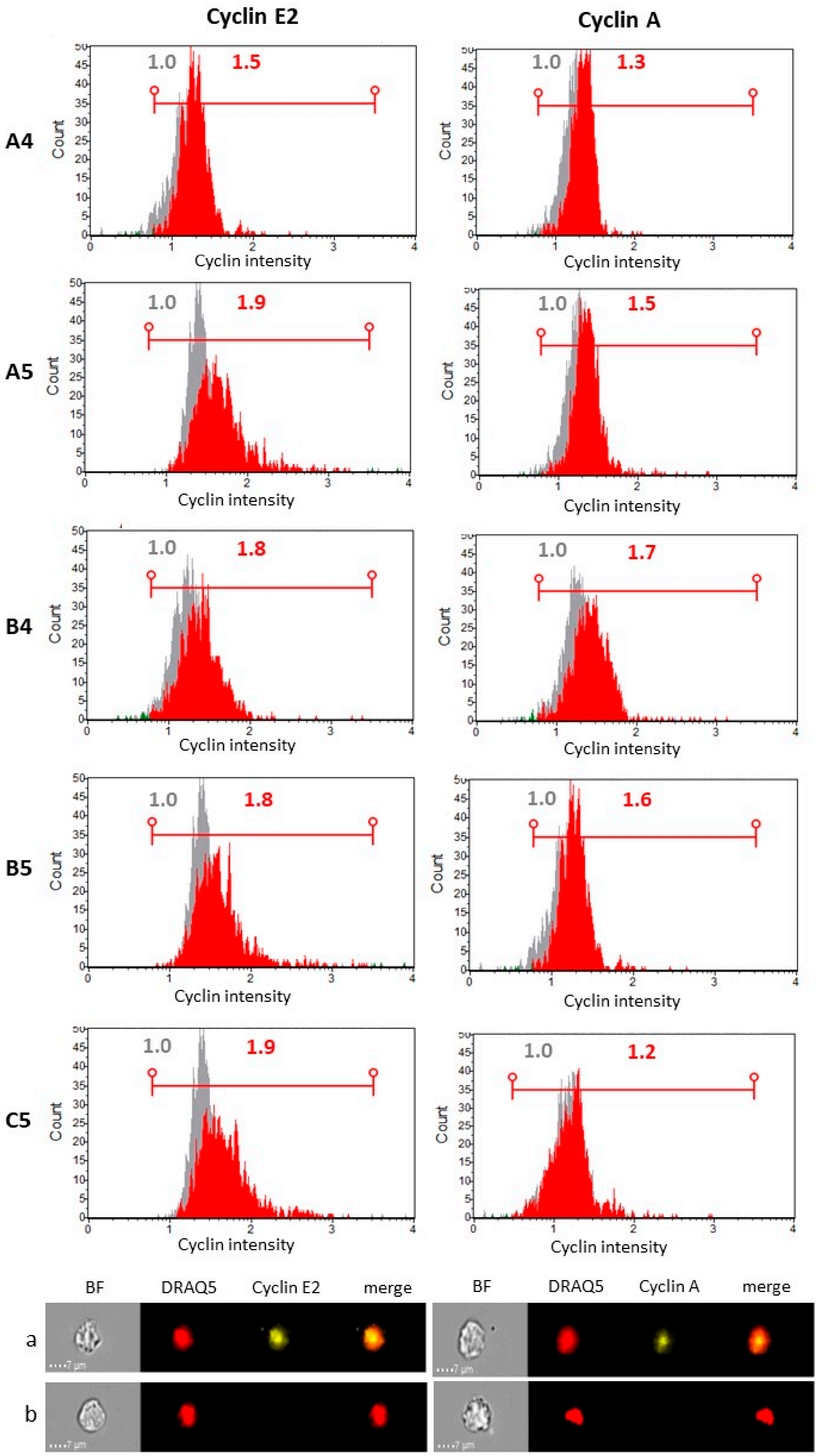
Cyclin E2
Cyclin A
A4
A5
B4
B5
C5
Count
Cyclin intensity
1.0
1.5
1.3
1.9
1.5
1.8
1.7
1.8
1.6
1.9
1.2
BF
DRAQ5
Cyclin E2
merge
Cyclin A
a
b

Figure S2. The concentrations reported in BIOPEP database, were further confirmed by a cytofluorimetric evaluation of Cyclins E2 and A expression in SUP-T1 cells

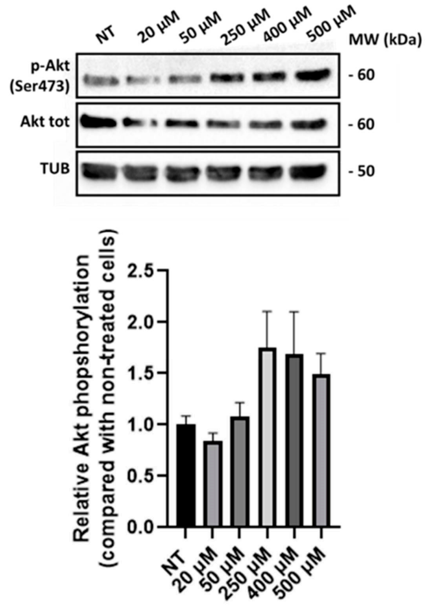

Figure S3. Immunoblotting analysis (upper panels) and relative densitometric analysis (lower panel) of Akt protein levels in Caco-2 cells treated with GEs (i.e., 20, 50, 250, 400, and 500 μM) after 4 hours p.t. Phosphorylated proteins were normalized to the total proteins and housekeeping levels. Normalized values are reported on Y-axis as arbitrary units. Molecular weights (MW) in kDa and SEM bars are reported. The experiments were performed on three independent biological replicas.

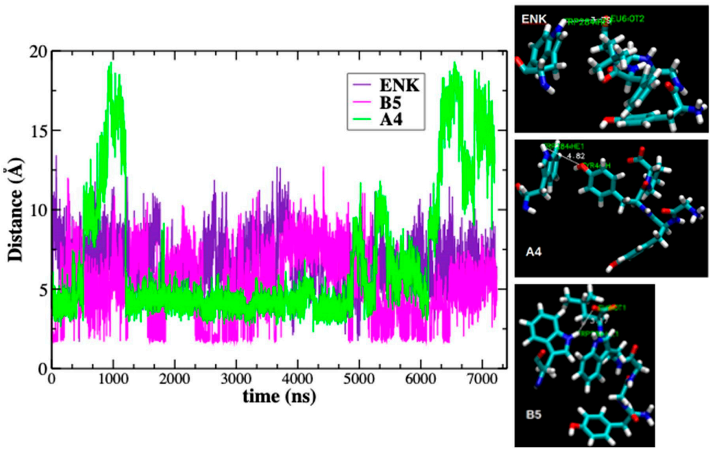

Figure S4. Timeline plot of atomic distance between the Trp384 side chain polar hydrogen and the proximal oxygen of the peptide ligand, either Tyr1 or the C terminal group (see right). The distance is calculated over a concatenated trajectory comprising the 6 independent runs (see Methods in main text for details). A4 shows a different behavior relative to ENK and B5, with a frequently increased distance corresponding to a loss of coordination in multiple trajectories.

Table S1. GEs amino acid sequences, molecular weight and purity.

Name	Sequence (Single letter code)	Molecular Weight	Purity
A4	[NH_2]GYYP[COOH]	498.53	> 99%
A5	[NH_2]GYYPT[COOH]	599.63	> 98%
B4	[NH_2]YGGW[COOH]	481.51	> 95%
B5	[NH_2]YGGWL[COOH]	594.67	> 95%

C5	[NH_2]YPISL[COOH]	591.71	> 99%
